# Assessing Patient Attitudes to Computerized Screening in Primary Care: Psychometric Properties of the Computerized Lifestyle Assessment Scale

**DOI:** 10.2196/jmir.955

**Published:** 2008-04-18

**Authors:** Farah Ahmad, Sheilah Hogg-Johnson, Harvey A Skinner

**Affiliations:** ^3^Faculty of HealthYork UniversityTorontoONCanada; ^2^Faculty of MedicineUniversity of Toronto and Institute of Work and HealthTorontoONCanada; ^1^Centre for Research on Inner City HealthSt. Michael’s HospitalTorontoONCanada

**Keywords:** Computers, scale, psychometric, screening, risk assessment, family practice

## Abstract

**Background:**

Computer-based health-risk assessments are electronic surveys which can be completed by patients privately, for example during their waiting time in a clinic, generating a risk report for the clinician and a recommendation sheet for the patient at the point of care. Despite increasing popularity of such computer-based health-risk assessments, patient attitudes toward such tools are rarely evaluated by reliable and valid scales. The lack of psychometric appraisal of appropriate scales is an obstacle to advancing the field.

**Objective:**

This study evaluated the psychometric properties of a 14-item Computerized Lifestyle Assessment Scale (CLAS).

**Methods:**

Out of 212 female patients receiving the study information at a family practice clinic, 202 completed a paper questionnaire, for a response rate of 97.6%. After 2 weeks, 52 patients completed the scale a second time.

**Results:**

Principal component analysis revealed that CLAS is a multidimensional scale consisting of four subscales (factors): (1) Benefits: patient-perceived benefits toward the quality of medical consultation and means of achieving them, (2) Privacy-Barrier: concerns about information privacy, (3) Interaction-Barrier: concerns about potential interference in their interaction with the physician, and (4) Interest: patient interest in computer-assisted health assessments. Each subscale had good internal consistency reliability ranging from .50 (2-item scale) to .85 (6-item scale). The study also provided evidence of scale stability over time with intraclass correlation coefficients of .91, .82, .86, and .67 for the four subscales, respectively. Construct validity was supported by concurrent hypotheses testing.

**Conclusions:**

The CLAS is a promising approach for evaluating patients’ attitudes toward computer-based health-risk assessments.

## Introduction

The use of computer interactive technology in health care settings is on the rise. Many studies report using patient-administered computer programs for health-risk assessments [1-4] as well as for preventative health education in clinical settings [5-7]. Interest is particularly growing in computer-based health-risk assessments for which patients complete a computer survey privately during their waiting time. The interactive program then prints a risk report for the clinician and a recommendation sheet for the patient at the point of care. Such computer-based health-risk assessments can facilitate meaningful communication between clinicians and patients by saving the clinicians’ screening time for thorough risk management and by allowing the patients to self-reflect on their risk profile before the medical consultation.

Many practical implications have also been recognized for computer-based health-risk assessments. At the organizational level, the advantages include speed and efficiency, accountability, quality improvement, and cost containment [8]. At the individual level, convenience to providers and patients includes tailored testing, accuracy of responses, unobtrusive means of branching or skipping questions, instant feedback on risks and referrals to clinicians and patients, and aids to diagnosis [9]. Technological advances, such as touch-screen, mobile, hand-held tablet computers, have amplified the utility of computer-based health-risk assessments. Such interactive computer technology has great potential in primary care settings where provider time is constrained due to a multitude of presenting health issues and preventive needs of patients [10].

However, user attitudes toward interactive computer technology are important when considering applications. In 1986, Nickell and Pinto developed a computer attitude scale for the general population [11]. Despite good psychometric properties [12], this scale has limited applicability in the physician-patient context due to specific communication patterns between health care providers and patients. While computer attitude scales have been developed and evaluated for physicians [13,14] and other health care providers [15], reliable and valid scales for general patients are lacking [16]. Further, little knowledge exists about scale reliability over time [16].

In our review of the literature on computer-based health-risk assessments, two scales were identified as potentially applicable to general patient populations. The first scale was developed by Lucas in 1977 and tested among patients visiting specialized clinics in hospital settings [17]. This 22-item scale tapped patient attitudes toward different types of clinical encounters, including computer-assisted visits, in-person visits, and ideal health care visits. The assessments used a semantic differential technique whereby participants rate each item on a bipolar scale with contrasting adjectives at the extremes, such as hot and cold [18]. The Lucas scale was subsequently used by others in a primary care setting [2]. Yet this scale is difficult to apply in today’s world of globalization, given different interpretations of adjectives by respondents of diverse ethnocultural backgrounds. Further, scales based on a semantic differential technique are lengthy and, hence, of limited use in time-pressed clinical settings.

Addressing some of these concerns, Skinner developed a short 14-item Computerized Lifestyle Assessment Scale (CLAS) in 1993 [19]. First drawing on an initial study of family practice patients [2], a large pool of items was generated through group discussions with patients and providers about the pros and cons of computer-based health-risk assessments. This list was reviewed by the research team, and the final pool of items was established through consensus among the team members. An easy to comprehend Likert-type scale was used whereby participants are asked to express their level of agreement or disagreement for each item. Given the centrality of decisional balance (ie, extent of pros compared to cons) in health behavior theories and research, the CLAS focuses on patient perceived benefits and barriers toward computer-based health-risk assessments. Several studies grounded in the Transtheoretical Model and Health Belief Model demonstrate that preventive behaviors, such as cancer screening visits, improve when perceived benefits exceed perceived barriers [20,21]. Thus, assessment of the decisional balance of patients in relation to computer-based health-risk assessments is meaningful in establishing their acceptance of future use. Although CLAS is a theoretically informed scale for primary care patients, its psychometric properties have not been previously reported.

Lack of psychometric appraisal of scales may impede research and innovation to advance the field. Recent studies have begun to report patients’ general reactions to the use of computer interactive technology. In 2000, Dugaw et al reported patients’ overall acceptance of computerized medical history taking in an emergency department, with limited description of the measurement [22]. Likewise, recent randomized trials on computer-based health-risk assessments by Rhodes et al in a US hospital emergency department reported general reactions of patients, their recall of advice after a 1-month follow-up, and satisfaction with the visit [3,4,23]. Although information on patient satisfaction is important, it does not generate knowledge specific to technologically mediated patient concerns or facilitators in medical encounters. In 2006, MacMillan and colleagues reported patients’ higher preference for computer-based screening for the risk of partner abuse compared to face-to-face inquiry. However, the preference measurement relied on three de novo questions about “ease,” “like answering,” and “private enough” [24]. The use of nonstandardized methods of measurement could lead to difficulties in assessing and interpreting results over time and across sites. At the same time, clinical adoption of computer-based health-risk assessments is dependent on the availability of reliable and valid knowledge about patient attitudes.

Considering the potential of CLAS, this study evaluated its psychometric properties as part of a larger research program on computer-based screening for lifestyle risks, including partner abuse, among female patients. Using standard procedures [25,26], the aim of this study was threefold: (1) to assess the dimensionality and/or latent constructs of CLAS, (2) to measure test-retest reliability and internal consistency of the instrument, and (3) to provide initial evidence on its construct validity.

## Methods

The study was conducted at a multidisciplinary family practice clinic affiliated with a teaching hospital in Toronto, ON, Canada. The study was approved by the hospital research ethics board as part of a research project on prevalence of partner abuse; details are provided elsewhere [27].

### Participant Recruitment

All adult female patients with an appointment were eligible to participate if they were at least 18 years of age, could speak and read English, and could provide informed consent. The study participants were recruited in 15 days over a period of three consecutive weeks in February of 2004. On recruitment days, all adult female patients with appointments were given a brief letter of invitation by the clinic receptionist at the time of arrival. These potential participants were then approached in the waiting area by a recruiter to confirm their eligibility and inquire about their interest in the study. Willing participants were taken to a separate room in the clinic, unaccompanied by family or friends, where they completed the survey after giving informed consent. At this time (T1), participants were also asked to consent to a subsequent contact after 2 weeks (T2) to administer the CLAS a second time. Participants sealed the survey in the provided envelope before returning it to the recruiter. Then, participants received health brochures (domestic violence, cancer, and heart health) with telephone numbers for domestic violence counsellors and the assaulted women’s helpline.

### Measurement

The survey included the CLAS, which is a 14-item scale that covers patients’ positive and negative perceptions about computer-based health-risk assessments [19]. Participants read a vignette about such a “computer survey” (Textbox 1) and rated each CLAS statement on a Likert-type scale of 1 to 5 (strongly agree, agree, not sure, disagree, strongly disagree). Other sections in the survey gathered information on sociodemographics (age, marital status, country of birth, years lived in Canada, highest education completed, employment status, and income), health (self-rated health, partner violence, and number of visits to family practice), exposure to computers (access and use), comfort in completing the survey, and English language abilities (see Table 1 for details).

**Vignette**

We would like to know your opinion about a computer survey of patients. This survey is completed by patients on a computer before seeing their family doctor. The computer survey asks questions about lifestyle and health risks such as smoking, stress, conflict in relationships, and safety. The questions appear on the computer screen one by one. The patient answers by touching one of the options on the computer screen using a non-ink pen. Patients do not type or use any computer parts but only touch the screen to give answers. This computer survey uses simple day-to-day language of 5th grade reading level. The computer system prints (1) a summary of patient health risks for the doctor to review, and (2) an information sheet for the patients about their reported health risks. *What is your opinion about such computer-based health-risk assessment of patients before seeing a family doctor*?

### Sample Size

The aim was to recruit a sample of 200 participants. As CLAS included 14 items, a sample of 200 was expected to generate an adequate subject-to-variable ratio of 14:1 to derive latent constructs. For factor analytical approaches, Gorsuch (1983) and others recommend a subject-to-variable ratio of five when the communalities are high and there are many variables for each factor [28,29]. If these conditions are not met, then a subject-to-variable ratio of 10 is recommended [28]. Others suggest that a sample of 150 should be considered sufficient when the factor analyzed solutions have several high loading markers (> 0.80) [30]. Our sample size is fair in meeting both of the established requirements (minimum sample size and sample size per item) for psychometric studies.

### Data Analyses

The CLAS items [19] were reverse coded prior to analyses so that 1 referred to “strongly disagree” and 5 to “strongly agree.” All analyses were conducted using Statistical Package for Social Sciences (SPSS) version 12(SPSS Inc, Chicago, IL, USA). Preliminary statistical procedures examined distributions of the individual items (eg, means, standard deviations, skewness, and kurtosis) and evidence of ceiling or floor effects. The quality of data was evaluated by percentage of missing responses, which were low; hence, we used the case deletion in subsequent analyses. The sampling adequacy was assessed by the Kaiser-Meyer-Olkin test.

Prior to reliability and validity analysis, we examined the latent structure of the scale. The latent constructs of the CLAS were examined by employing principal component analysis (PCA) [31,32]. The number of latent constructs or factors was determined using scree plots and the criterion of eigenvalues greater than 1.0. We considered three-factor, four-factor, and five-factor solutions with varimax rotation. Salient loadings were defined using a critical value of 0.38 [28].

The scale reliability was estimated by both internal consistency and test-retest reliability of the subscales. To examine homogeneity of items or internal consistency, item-total correlation [33] and change in Cronbach alpha coefficient upon item deletion were used [34]. For test-retest reliability, intraclass correlation (ICC) from a two-way random effects model was executed [35]. We also compared T2 participants to the remaining participants at T1 with respect to characteristics measured at T1 to assess the potential to generalize the reliability findings.

After factors were derived and reliability established, construct validitywas investigated. For this analysis, we tested hypotheses that were based on existing literature. Further details on the hypotheses are presented in the Results section under construct validity. The hypotheses were tested by using Pearson product moment (*r*_p_), point biserial (*r*_pb_), or Spearman rank (*r*_s_) correlation analyses, as appropriate.

## Results

### Participants

Among 361 women approached, 212 eligible women received the study details in privacy, 207 provided written consent (response rate 97.6%), and 202 returned the completed surveys. Participants had a mean age of 45.3 years (range 19 to 86) and 36% were immigrants, with the top two groups from Europe and Asia (Table 1). Almost 75% of the participants were in a current intimate relationship, and 77% had at least university education. Nearly 64% were currently employed and reported annual household income of at least Can $40,000; 87% of the participants had access to computers, and 66% used one every day. Self-perceived health was rated as “good” on a scale of one to five with a mean of 3.2 (SD 1). The mean number of visits to the family practice during the last year was 4.6 (median 3.5; mode 1; range 0 to 30).

**Table 1:** Sociodemographic characteristics (N = 202)

Variable	No.	%
Age (years), mean (SD)	201	45.3 (15.4)
Current marital status	202	
Married or common law or intimate		74.9
Separated or divorced or widowed		13.9
Single, not in relationship		11.4
Country of birth: Canada	129	63.9
If immigrant: years lived in Canada	71	
Less than 10 years		23.9
11 to 20 years		16.9
More than 20 years		59.2
If immigrant: country of birth	72	
Europe		36.1
East or South East Asia or South Asia		29.1
West Indies, Latin America, or Caribbean		20.8
Middle East or West Asia		6.9
Africa		5.6
Highest education	201	
Less than high school		3.0
High school, some or complete		19.9
University or higher, some or complete		77.1
Current employment	201	
Full-time or part-time		64.2
Unemployed		13.9
Retired or on disability		21.9
Household annual income (Can $)	181	
Less than 20,000		15.5
20,001 to 40,000		19.9
40,001 to 60,000		20.5
More than 60,000		44.2
Access to computer at home or work	200	87.0
Use of computer in the last month	200	
Every day or two to three times a week		81.5
Once a week or once a month		6.5
Not at all		12.0
English ability,^*^ mean (SD)	201	4.5 (0.87)
Survey comfort level,^†^ mean (SD)	199	4.0 (1.2)

^*^Scale of 1 to 5: 1 = poor, 2 = fair, 3 = good, 4 = very good, 5 = excellent.

^†^Scale of 1 to 5: 1 = very uncomfortable, 2 = uncomfortable, 3 = not sure, 4 = comfortable, 5 = very comfortable.

**Table 2:** Item summary statistics and Pearson correlations

Item^†^	% Miss^*^	Mean^‡^	SD	Skewness	Kurtosis	Item Correlation													
						1	2	3	4	5	6	7	8	9	10	11	12	13	14
1. Routine	1.0	3.93	0.86	-0.74	0.62	1													
2. Lifestyle	0.5	3.67	0.94	-0.73	0.39	.58	1												
3. Save time	2.0	3.73	0.92	-0.64	0.55	.62	.47	1											
4. Better assess	1.0	3.28	0.84	-0.07	0.17	.54	.43	.50	1										
5. Comfortable	1.0	3.92	0.85	-0.86	0.93	.60	.52	.41	.34	1									
6. Trusted	2.0	3.36	0.94	-0.33	0.02	.47	.33	.37	.42	.59	1								
7. Confidentiality	0.5	3.30	1.14	-0.17	-1.06	-.08	-.05	-.80	-.06	-.24	-.29	1							
8. Certain information	0.5	3.39	1.12	-0.24	-0.78	-.14	-.04	-.15	-.04	-.22	-.26	.47	1						
9. Mistakes	1.0	2.63	0.84	0.51	0.27	-.18	-.16	-.14	-.27	-.39	-.41	.41	.38	1					
10. Less time	2.0	3.35	0.99	-0.34	-0.41	-.06	.02	.16	-.02	-.19	-.13	.25	.17	.26	1				
11. Personal touch	1.0	3.38	1.19	-0.14	-1.12	-.32	-.24	-.20	-.30	-.34	-.32	.28	.40	.43	.40	1			
12. Another doctor^§^	0.5	2.13	0.98	0.98	0.92	-.35	-.28	-.30	-.33	-.47	-.27	.29	.26	.42	.32	.50	1		
13. Answer honestly^§^	1.5	4.42	0.68	-1.36	3.36	.40	.19	.32	.23	.50	.28	-.07	-.19	-.20	-.12	-.20	-.35	1	
14. No pat info^§^	4.5	4.26	0.70	-0.97	2.15	.35	.19	.19	.20	.25	.23	-.04	-.03	-.06	-.06	-.06	-.12	.34	1

^*^% Miss, % missing response.

^†^Full item statements are provided in Table 3 and Table 4.

^‡^Scale of 1 (strongly disagree) to 5 (strongly agree).

^§^Skewed items.

### Item Descriptive Statistics

The item means and standard deviations were acceptable, while three items were skewed (Table 2). These items were transformed and PCA was executed with and without transformations. As the two PCAs were similar in factor structure and factor loadings, we report PCA without transformed items in this paper. Sampling adequacy was indicated by a Kaiser-Meyer-Olkin test value of .82 and the absence of ceiling or floor effects. The factorability was indicated by correlation and partial correlation matrices.

### Factor Structure

On conducting the PCA, the first 10 eigenvalues were 4.7, 2.1, 1.1, 1.0, .85, .76, .69, .53, .50, and .44. Four factors emerged with eigenvalues greater than or equal to one, accounting for 63.7% of the total variance. Based on the scree plot, either a three-factor or four-factor solution was indicated. We considered three-, four-, and five-factor solutions, and the four-factor solution yielded the most interpretable results. A summary of the PCA with varimax rotation is presented in Table 3. The factors were named Benefits, Privacy-Barrier, Interaction-Barrier, and Interest. Three- and four-factor solutions were also compared for the internal consistency of the derived subscales. Although the Privacy-Barrier and Interaction-Barrier factors merged into one factor upon forcing a three-factor solution, the internal consistency of the subscales was higher in the four-factor solution than in the three-factor solution. This internal consistency comparison was based on the reliability coefficients adjusted for the length of the subscales [33].

**Table 3:** Summary of principal component analysis with varimax rotation

Item	Factor Loadings				*h*^2*^
	Benefits	Privacy -Barrier	Interaction -Barrier	Interest	
1. Computers will help doctors with routine lifestyle questions	.79				.74
2. The computer is a good way to ask lifestyle questions	.79				.65
3. It would save doctors time.	.78				.65
4. Doctors will make better assessments with such computer systems	.74				.60
5. I would feel comfortable answering questions on a computer	.58				.61
6. Computers can be trusted	.54	(.41)^†^			.51
7. I would worry about confidentiality		.82			.69
8. I do not want certain information about me on the computer		.81			.69
9. Too many mistakes will be made with computer		.60	(.39)^†^		.55
10. Doctors would spend less time with patients			.81		.71
11. There will be loss of personal touch of a doctor			.69		.67
12. I would find another doctor			.63		.57
13. I would want to read patient information sheet				.80	.67
14. I would answer honestly				.74	.62

*h ^2^ refers to communalities.

^†^Item shared loading between factors above the critical value.

Variances accounted for by the four identified factors (Benefits, Privacy-Barrier, Interaction-Barrier, and Interest) after the rotation were 33.6%, 15.0%, 8.0%, and 7.2%, respectively. The item “Computers can be trusted” in the first factor (Benefits) shared loading (.41) with the second factor (Privacy-Barrier) above the critical value of .38. Also, the item “Too many mistakes will be made with computer” in the second factor (Privacy-Barrier) shared loading (.39) with the third factor (Interaction-Barrier) above the critical value.

The Benefits factor consisted of six items with factor loadings ranging from .79 to .54. The items loading on this factor cover perceived benefits toward the quality of medical consultation and means of achieving the benefits. The Privacy-Barrier factor consisted of three items dealing with patient concerns about privacy, with loadings ranging from .82 to .60. The Interaction-Barrier factor consisted of three items covering patient concerns about interference in the interaction with the physician, with loadings ranging from .81 to .63. Although the Interest factor consisted of only two items, both items had strong factor weightings (ie, .80 and .79). The stability of this factor was also apparent during execution of the five-factor solution. Both items of this factor continued to load together while the fifth factor consisted of one item pulled from the Interaction-Barrier factor.

### Reliability

To estimate internal consistency reliability, we considered the following criteria for each subscale: (1) an item-total correlation of at least .3 for all items, (2) no increase in the Cronbach alpha coefficient if an item was deleted, and (3) general acceptability of the item means and standard deviations. All three criteria were met for the subscales (Table 4).

**Table 4:** Internal consistency of the subscales

Item	Mean (SD)^*^	Corrected Item-Total Correlation	Cronbach Alpha if Item Deleted
**Benefits: Cronbach alpha .85**			
1. Computers will help doctors with routine lifestyle questions	3.9 (0.87)	.77	.80
2. The computer is a good way to ask lifestyle questions	3.7 (0.94)	.62	.83
3. It would save doctors time.	3.7 (0.91)	.61	.83
4. Doctors will make better assessments with such computer systems	3.3 (0.85)	.59	.83
5. I would feel comfortable answering questions on a computer	3.9 (0.85)	.66	.82
6. Computers can be trusted	3.4 (0.95)	.57	.84
**Privacy-Barrier: Cronbach alpha .70 (alpha .81^†^)**			
7. I would worry about confidentiality	3.3 (1.1)	.53	.54
8. I do not want certain information about me on the computer	3.4 (1.1)	.52	.55
9. Too many mistakes will be made with computer	2.6 (0.84)	.46	.64
**Interaction-Barrier: Cronbach alpha .67 (alpha .80^†^)**			
10. Doctors would spend less time with patients	3.4 (0.99)	.42	.66
11. There will be loss of personal touch of a doctor	3.4 (1.2)	.56	.47
12. I would find another doctor	2.1 (0.97)	.49	.57
**Interest: Cronbach alpha .50 (alpha .75^†^)**			
13. I would want to read patient information sheet	4.3 (0.70)	.34	–
14. I would answer honestly	4.4 (0.63)	.34	–

^*^Scale 1 to 5: strongly disagree, agree, not sure, agree, strongly agree.

^†^Adjusted reliability coefficient, adjusted to compare to scales with six items.

The Cronbach alpha coefficients for the four subscales Benefits, Privacy-Barrier, Interaction-Barrier, and Interest were .85, .70, .67, and .50, respectively. There was no increase in Cronbach alpha if items were deleted from the first three subscales. This analysis did not apply to the Interest subscale as it had two items only. The item-total correlation for the subscales Benefits, Privacy-Barrier, and Interaction-Barrier ranged from .77 to .57, .53 to .46, and .52 to .44, respectively. We also calculated the reliability coefficients adjusted for the length of subscale [33], given that the number of items loading on the subscales varied and that Cronbach alpha is sensitive to number of items. The adjusted reliability coefficients were .81, .80, and .75 for the Privacy-Barrier, Interaction-Barrier, and Interest subscales, respectively, where adjustment was made to assume six items as for the Benefits subscale. This analysis assumes that the new items would be similar to the old items with respect to content and reliability.

Scale reliability over time was assessed with the test-retest data (n = 52). At T2, 52 patients were successfully reached out of 145 T1 participants who consented to the second contact. The reduced participation at T2 was due to (1) the study requirement that the second administration of the CLAS occur within 2 weeks of the first administration, and (2) the fact that many patients were difficult to reach because they had provided telephone numbers at work. The T2 participants were similar to the other T1 participants (n = 150) on sociodemographic characteristics, including age, country of birth, number of years lived in Canada, education level, employment status, income, English language abilities, access to computers, computer use in the last month, relationship status, experiences of intimate partner violence, number of visits to family practice, and perceived health. However, the T2 participants were less likely to be employed than participants who consented but could not be reached for second contact (*χ* ^2^_2_= 7.0, *P* < .05). The time between T1 and T2 contacts averaged 16 days (SD 2.6, median 15, mode 15). The ICC analysis based on a two-way random effect model gave coefficients of .76 for the overall scale and .91, .82, .86, and .67 for the subscales of Benefits, Privacy-Barrier, Interaction-Barrier, and Interest, respectively. As the CLAS is a multidimensional scale, the test-retest reliability of the subscales was higher than the test-retest reliability of the overall scale.

### Construct Validity

To evaluate validity of the derived constructs, several hypotheses were formulated based on a literature review. We hypothesized that the Benefits factor would be positively associated with participants’ frequent use of computers as greater familiarity with computers is likely to increase peoples’ comfort and perceptions of the benefits [2]. Also, we hypothesized that patients with poorer health would perceive the benefits of computer-based screening as high due to the limited time available for lifestyle inquiries during their routine health care visits. As computer-based screening has been found specifically beneficial for socially sensitive issues [2-4,36-39], it was hypothesized that participants reporting victimization by intimate partners would perceive the benefits as high. Existing studies report that patients are likely to perceive barriers in using preventive health services if they have low socioeconomic status or are immigrants [40,41]. Accordingly, it was hypothesized that the Privacy-Barrier and Interaction-Barrier factors would be positively associated with participants’ non-Canadian-born status, low household income, unemployment, and lesser years of education. We also hypothesized that the Interest factor would be significantly associated with less use of computers and older age.

The hypotheses were tested by correlation analyses. The Benefits factor was positively associated with poorer self-perceived health and intimate partner victimization (*r*_p_ = .15, *P* = .03; *r*_pb_= .19, *P* = .02) as hypothesized. However, it was not significantly associated with the use of computers, in contrast to our hypothesis. To explore further, we examined the mean scores of the Benefits subscale by participants’ frequency of computer use in the last month. Participants who used computers every day or two to three times a week somewhat agreed with the Benefits (mean 3.7, SD 0.67), while participants who used computers once a week or once a month (mean 3.5, SD 0.50) or not at all (mean 3.6, SD 0.67) seemed to neither agree nor disagree with the Benefits.

As hypothesized, the Privacy-Barrier and Interaction-Barrier factors had positive significant associations with participants’ non-Canadian-born status (*r*_pb_= .19, *P* = .006; *r*_pb_= .22, *P* = .001), low household income (*r*_p_= .23, *P* = .002; *r*_p_ = .21, *P* = .004), and lower use of computers (*r*_s_ = .16, *P* = .03; *r*_s_ = .18, *P* = .01). Furthermore, older age at the time of immigration had a positive association with both the Privacy-Barrier and Interaction-Barrier factors (*r*_p_ = .27, *P* *=* .02; *r*_p_ = .28, *P* = .02). The Interaction-Barrier factor also had significant associations with participants’ unemployment status and lesser years of education (*r*_pb_ = .16, *P* = .03; *r*_s_ = .18, *P* < .01). The Interest factor had significant positive associations with older age (*r*_p_ = .16, *P* = .03) and less use of computers (*r*_s_ = .14, *P* = .04).

## Discussion

The CLAS has demonstrated good preliminary psychometric properties and shows promise as a tool for assessing patient attitudes toward computer-based health-risk assessments. Each of the four latent constructs or derived subscales of the CLAS had good internal consistency that exceeded the recommended threshold of 0.7 [42] after adjusting for the number of items. Furthermore, the multidimensionality of the CLAS highlights different clusters of barriers perceived by patients in the use of interactive technology, namely privacy and interaction with physicians. This study also provides much needed initial evidence of the scale stability over time through test-retest analysis. This is important as some researchers and health care interventionists aim to assess patient attitudes toward computer-based health-risk assessments before and after new initiatives.

### Implications

The use of a psychometrically validated scale is an essential element in facilitating clinical and policy decisions about the application of computer-based health-risk assessments. This is of particular importance for sensitive health risks and conditions where superiority of computer-based risk assessments over personal interviews is already well documented with respect to patient disclosure of socially sensitive information. These health risks and conditions include behaviors related to sex, alcohol, drugs, HIV, and violence [2-4,36-39]. A similar link is demonstrated in our study as a positive association between women’s victimization at the hands of their intimate partner and the Benefits subscale. Literature shows that women experiencing partner abuse seldom spontaneously disclose it to health care providers [43,44], who frequently fail to detect victims of abuse due to time pressure, priority of acute problems, and discomfort [45,46]. At the same time, clinicians’ questioning about abuse is the most significant predictor of women’s disclosure [47]. Computer-based screening matches abused women’s preferences for “direct questioning,” and it has limited dependency on physician time. Above all, it is a nonjudgmental and anonymous way of asking about socially sensitive health risks. Perhaps it explains why abused women in our study perceived higher benefits of the computer-based screening. Our future work will test the computer-based screening intervention in a family practice setting for the detection and disclosure of partner abuse.

The findings also highlight the complex nature of human behavior. Study participants perceived barriers in two distinct ways: barriers regarding privacy and barriers regarding interaction with physicians. At the implementation level, this underscores the need to measure both domains to understand and thereafter address effective use of computer-based health-risk assessments. At the theoretical level, this distinction is novel to the original conception of the scale. Possibly, patient attitudes have taken specific forms with the increasing use of computers. Recent studies reveal that use of the Internet for health information influences the way people relate to physicians, make medical decisions, and access health services [48,49]. In 2007, a telephone survey with 2479 Canadians examined their attitudes toward electronic health information and their privacy [50]. The survey found that 9 out of 10 people perceived the use of electronic health information as integral to the provision of high-quality care but had mixed confidence about the protection of health information. Future research should further examine the domains of privacy and interaction barriers in the use of computer-based health-risk assessments. Other studies report that patients’ perceptions toward computer-based lifestyle assessments are positively increased after they are provided the actual experience [2,19].

Our post hoc analyses indicate that study participants who were immigrants or had lower socioeconomic status perceived more barriers. This raises two critical questions: (1) Is this an extension of the “digital divide?”, and (2) What does it mean for implementation? The term “digital divide” stems from research and refers to “decreased access to information technologies, particularly the Internet, for racial and ethnic minorities, person with disabilities, rural populations, and those with low socioeconomic status” [51] (p 449). The digital divide requires vigilance when using certain health information technologies, such as the Internet [52,53]. In contrast, computer-based health-risk assessments in health care settings bridge the digital divide because these programs provide tailored health information to the patients at the point of care. They may play a positive role in addressing patients’ unequal access to health information and care—an anticipated impact similar to telemedicine [54].

### Limitations

Several limitations of this study should be noted. The CLAS predominantly measures the decision-making aspect of human behavior, though it has relevance for research on explaining and changing behavior regarding computerized assessments. Future studies should explore other aspects such as patient self-efficacy and cue-to-action. The construct of Interest would also benefit from further conceptual development. Further, our analysis of the construct validity is post hoc in nature. Many of the correlations were not strong even when significant. This is possibly due to our convenience-based use of a larger survey to select variables which in turn had a more distal than proximal relationship with the CLAS constructs. Although we found support for most of our hypothesized relationships, the Benefits subscale was not associated with the participants’ use of computers, contrary to our hypothesis. The study sample was relatively more educated than the average general population, and 87% of the participants had access to computers at home or work; almost a similar proportion reported using the computer every day or at least two to three times a week. Perhaps frequent use of computers makes people think critically about their advantages and disadvantages, leading to a cautious assessment of their benefits. On other side, it is also possible that computers have now become part of our everyday life and their benefits are taken for granted, reducing the level of perceived benefits seen a few years ago. Future research with larger samples should examine this further and establish the construct validity with a priori selection of variables. Also, it will be important to conduct a classic multitrait-multimethod study in which the four constructs on the CLAS are assessed via different methods (eg, peer ratings, behavior observations). This type of study will provide evidence for both convergent and discriminate aspects of the CLAS construct validity.

Caution is warranted regarding the generalizability of our study findings. We evaluated psychometric properties of the CLAS with female patients only. A future study involving both men and women is needed to ensure its applicability to all patients visiting primary health care settings. Further, patients were recruited from a single site. However, the collaborating clinic had several physicians and served a large number of diverse patients with estimated annual visits of 50,000. The study obtained a high response rate and, reassuringly, the participants were similar to females residing in Toronto in terms of immigration and marital status [55,56]. At the same time, study participants had relatively higher levels of income and education than the general population. The test-retest results of our study may have limited generalizability as participants in the second administration of the CLAS were more likely to be unemployed than the rest of participants. Nevertheless, the two groups were similar for all other sociodemographic and health-related variables that were measured. Further research is needed with a heterogeneous sample as an important next step to advance the generalizability of the scale.

### Conclusion

This study of patients in a family practice setting advances our understanding of the properties, applicability, and generalizability of the CLAS. This is an important improvement over previous assessments of other scales that relied on samples of convenience or were not specific to patient populations. Furthermore, the phrasing of items in the CLAS is expected to allow people from different ethnocultural backgrounds to reply in a meaningful way, unlike some other existing scales. At the same time, future research with a heterogeneous sample is needed to enhance its generalizability by gender and socioeconomic status while examining the utility for low and high users of computers. In conclusion, this study is a step toward facilitating research and interventions for promoting patient acceptance of computer interactive technology.

## Abbreviations

CLAS: Computerized Lifestyle Assessment Scale
ICC: intraclass correlation
PCA: principal component analysis
